# The Impact of Perceived Neighborhoods in Childhood and Adulthood on Daily Cognitive Function

**DOI:** 10.1093/geroni/igaa057.2243

**Published:** 2020-12-16

**Authors:** Elizabeth Munoz, Giancarlo Pasquini, Alexa Allan, Jacqueline Mogle, Martin Sliwinski, Stacey Scott

**Affiliations:** 1 The University of Texas at Austin, Austin, United States; 2 Stony Brook University, Stony Brook, New York, United States; 3 Pennsylvania State University, University Park, Pennsylvania, United States; 4 Penn State University, University Park, Pennsylvania, United States

## Abstract

Neighborhood experiences may have a cumulative effect on cognitive health outcomes across the lifespan. We investigated independent and combined associations between current and retrospective childhood neighborhoods and daily cognitive function in 209 adults (Mage=47.07; range: 25-65) who participated in a 14-day ecological momentary assessment study. Participants reported perceptions of their current neighborhood and the neighborhood they lived in at age 5; including neighborhood cohesion, safety, violence, and physical conditions. Greater current neighborhood violence was independently associated with poorer spatial working memory. Childhood neighborhood violence was not significantly associated with performance. The interaction between current and childhood neighborhood ratings was significant: individuals who reported greater childhood neighborhood violence, but lower current violence had better performance than those who experienced consistently high or low neighborhood violence. Effects for other neighborhood domains were not significant. Results indicate that neighborhood influences on cognition in adulthood may be moderated by childhood neighborhood experiences.

